# Virtual Surgical Planning for Temporomandibular Joint Reconstruction with Stock TMJ Prostheses: Pilot Study

**DOI:** 10.3390/medicina60020339

**Published:** 2024-02-19

**Authors:** José Luis del Castillo Pardo de Vera, José Luis Cebrián Carretero, Íñigo Aragón Niño, Marta María Pampín Martínez, José Tadeo Borjas Gómez, Ignacio Navarro Cuéllar, Ana María López López, Estela Gómez Larren, Carlos Navarro Vila, Pablo Montes Fernández-Micheltorena, Álvaro Pérez Sala, Carlos Navarro Cuéllar

**Affiliations:** 1 Oral and Maxillofacial Surgery Department, University Hospital La Paz, Paseo de la Castellana, 261, 28046 Madrid, Spain; josel.cebrian@salud.madrid.org (J.L.C.C.); inigo.aragon@salud.madrid.org (Í.A.N.); martamaria.pampin@salud.madrid.org (M.M.P.M.); jose.borjas@idipaz.es (J.T.B.G.); 2 Oral and Maxillofacial Surgery Department, University Hospital Gregorio Marañón, C/Dr. Esquerdo 46, 28007 Madrid, Spain; ignacio.navarro@salud.madrid.org (I.N.C.); alopezl@salud.madrid.org (A.M.L.L.); egomezlarren.externo@salud.madrid.org (E.G.L.); cnavarrov@salud.madrid.org (C.N.V.); cnavarroc@salud.madrid.org (C.N.C.); 3 Oral and Maxillofacial Surgery Department, University Hospital San Pedro, C/Piqueras 98, 26006 Logroño, Spain; pmontesf@riojasalud.es (P.M.F.-M.); aperez@riojasalud.es (Á.P.S.)

**Keywords:** stock TMJ prosthesis, 3D virtual surgical planning, CAD/CAM surgical guides, TMJ reconstruction

## Abstract

The temporomandibular joint (TMJ) is one of the most complex joints in the human anatomy. In advanced degenerative stages, conservative or minimally invasive surgical therapies have failed to restore joint function, and joint replacement with prostheses has been required. Stock prostheses, compared to custom-made prostheses, are much less expensive and require less pre-operative preparation time. Four patients followed for years for temporomandibular dysfunction and previously operated on by arthroscopy or open joint surgery that have been reconstructed with stock TMJ prostheses (STMJP) through virtual surgical planning (VSP) and an STL model with surgical and positioning guides were included. The median follow-up was 15 months; the median number of previous TMJ surgeries was 2. The mean preoperative MIO was 24.6 mm and at longest follow-up was 36.4 mm. The median preoperative TMJ pain score was 8, and the median postoperative TMJ pain was 3. All patients have improved their mandibular function with a clear improvement of their initial situation. In conclusion, we believe that stock TMJ prostheses with virtual surgical planning and surgical guides are a good alternative for TMJ reconstruction at the present time. Nonetheless, prospective and randomized trials are required with long-term follow up to assess their performance and safety.

## 1. Introduction

Indications for temporomandibular joint (TMJ) reconstruction include bony ankylosis, degenerated or resorbed joints resulting in severe anatomic discrepancies, previous failed alloplastic and/or autogenous joint replacement, severe inflammatory joint disease that has failed conservative measures, post-traumatic injury, post-tumor reconstruction, and developmental anomalies [1,2,3].

In advanced degenerative stages of TMJ, open surgery (meniscectomy, discectomy, interposition of autologous tissues) does not always improve joint function, and sometimes, joint replacement with a joint prosthesis is necessary [4].

Numerous surgical techniques have been employed for decades to reconstruct mandibular defects, encompassing the mandibular condyle. These techniques include osteochondral grafts, microsurgical reconstructions, mandibular distraction, reconstruction plates, and mandibular condyle prostheses. Despite the longstanding use of autologous grafts, they have not entirely rectified mandibular defects nor fully restored joint function [5,6,7].

Unlike the use of autografts, prosthetic TMJ reconstruction offers several advantages. These include the restoration of jaw function, the absence of donor site morbidity, the potential for correcting skeletal deformities and malocclusions simultaneously, a significant reduction in surgical time, extensive reconstruction capabilities for joint defects, shortened hospital stay, and increased predictability of the final outcome [8].

The successful use of TMJ prostheses requires correct 3D preoperative planning, proper surgical technique, a good understanding by the patient of the expected results, adequate postoperative physiotherapy and close follow-up.

At present, there are two types of temporomandibular prostheses: stock and customized prostheses. The latter have the advantage of achieving a better adaptation to the area of the mandibular ramus and the glenoid cavity and zygomatic arch and present a design adapted to the articular biomechanics of the mandible in each patient. However, they have the disadvantage of a higher economic cost and require at least 3–4 months for their design and distribution by most commercial companies [9].

Nowadays, the integration of 3D virtual planning and the utilization of cutting and positioning guides for stock prostheses offer a means to enhance their adaptation to both mandibular and cranial anatomy. This not only reduces the economic cost but, more importantly, enables immediate surgery for patients experiencing substantial pain and limited jaw opening, as these prostheses can be made available within a few [10,11].

The purpose of this pilot study was to evaluate the outcomes of the reconstruction of TMJ with stock TMJ prostheses through virtual surgical planning (VSP), STL models, and surgical and positioning guides.

## 2. Materials and Methods

### 2.1. Patients

To address the research purpose, the investigators performed a retrospective study including patients with TMJ degenerative situation that had been reconstructed with stock TMJ prostheses at Hospital Universitario La Paz (Madrid, Spain) between 2020 and 2023 (Table 1). 

There were four women with a mean age of 48.7 years (range 42–68) and in only one case did the patient have a unilateral custom-made prosthesis fitted 5 years ago.

### 2.2. Preoperative Planning

The surgery was planned with the help of high-resolution computed tomography using 0.5 mm thin slices, and plaster models were used to plan the optimal position of the dental crowns.

The Dicom files obtained from the CBCT were imported into software to create three-dimensional (3D) models of the bone anatomy of each patient. Each file obtained was saved in STL format and then merged with the STL file obtained from the intraoral scanner and the diagnostic wax-up to improve all the patient’s bone and dental information and achieve greater precision in the final result of occlusion. The initial phase involved achieving optimal digital occlusion, which might necessitate adjusting the opposite side through a sagittal ramus osteotomy in cases involving unilateral prostheses. Subsequently, the positioning of osteotomies in the mandibular ramus was determined based on the desired final placement of the stock prosthesis, following confirmation of its accurate adaptation to the bone.

After confirming the accurate position and size of both components of the prosthesis, custom cutting and positioning guides are designed using CAD-CAM technology. A stereolithographic model of the case is then created to ensure the proper adaptation of splints and guides. In our approach, we employ a unified guide for both the branch and fossa, aligning it with the patient in the initial splinted occlusion, coinciding with the planning CBCT. This method enhances the precision of prosthesis placement.

Sometimes it is necessary to carry out a small remodelling in some areas to achieve perfect adaptation of the prosthesis to the bone, both in the area of the mandibular angle and in the area of the temporal fossa eminence.

The prostheses used from Walter Lorenz (Zimmer Biomet Inc., Biomet Microfixation Inc., Jacksonville, FL, USA) are stock prostheses in both components. The mandibular component is made from cobalto-chromium–molybdenum with a titanium alloy coating. It is available in three lengths and three styles (standard, narrow, and offset). The fossa component is made from ultra-high-molecular-weight polyethylene. The fossa is offered in three sizes. The fossa component is attached to the zygomatic arch with self-tapping screws 2.0 mm in diameter; the mandibular component is attached to the mandibular ramus with self-tapping screws 2.7 mm in diameter.

### 2.3. Surgical Procedure

#### 2.3.1. Preoperative Management

All procedures were performed under general anesthesia and nasotracheal intubation to allow opening and closing of the oral cavity and to check the patient’s occlusion. A single injection of 2 g of amoxiclavulanic acid was administered intraoperatively. 

Given the necessity for thorough asepsis during joint surgery, one surgeon is responsible for preparing the surgical field, while another focuses on operating on the intraoral area. Following the initial procedure, both surgeons undergo a fresh round of surgical handwashing and change into new gowns and gloves.

After infiltration with saline and vasoconstrictor (1:200.000 epinephrine) at the approach sites, an adhesive drape is applied. A 3M™ Ioban™ 2 antimicrobial adhesive drape is used to provide a sterile surface at the wound edge at the start of surgery and continuous antimicrobial activity throughout the procedure.

#### 2.3.2. Surgical Procedure

Two skin incisions are made, one preauricular and one submandibular, about 2 cm below the mandibular border to ensure a good surgical field that allows resection of the mandibular condyle, introduction of the surgical guides, and placement of the two parts of the prosthesis.

Once the approach to the mandibular condylar and zygomatic arch has been made, the cutting and positioning guides for the prostheses are introduced. At this point, an intermaxillary block is applied in the mandibular centric position as the CBCT was performed.

It must be verified that the adaptation of the prostheses to the bone is the same as in the planning and stereolithographic models. The osteotomy lines are marked with an ultrasonic surgical device both in the condyloid and in the eminence. Additionally, the positions of the screws, crucial in determining the placement of the prostheses, are marked.

The medial aspect of the mandibular condyle is protected at all times by means of a periostotome to avoid damage to the soft tissues. Usually, several osteotomies are performed to achieve removal of the entire mandibular condylar fragment, which facilitates its removal.

Once the condilectomy has been performed, the articular meniscus is removed, taking special care to coagulate the insertion of the lateral pterygoid muscle and to ream the entire bony surface of the joint to remove the fibrocartilage. Coronoidectomy is selectively undertaken, dependent on whether it poses interference with any component of the prosthesis; it is not performed universally in all cases.

Sometimes it is necessary to carry out minor remodeling in some areas to achieve perfect adaptation of the prosthesis to the bone, both in the area of the mandibular angle and in the area of the temporal fossa eminence. 

Once the osteotomies are completed, the surgical site is covered with a sterile adhesive dressing to avoid contamination of the surgical site. The oral cavity is then accessed, and a wire intermaxillary block is performed using the final splint. Despite the patient wearing orthodontics, the stability of the occlusion is further enhanced through the application of intermaxillary blocking screws. After this step, all instruments utilized within the oral cavity are carefully removed.

Surgeons who have performed the intermaxillary block remove gown and gloves to wash again, and the mouth is covered with new adhesive drapes to avoid cross-contamination of the oral cavity.

The adhesive dressing is then opened in the articular area, and a thorough cleansing with physiological saline solution and antibiotics is performed. The preauricular area is prepared and the prosthetic part of the fossa is placed, avoiding contact with the skin. The prosthesis is inserted into the glenoid cavity confirming the intended position and is held in place with the aid of a periosteal screw, avoiding manipulation of the prosthesis. It is securely affixed to the zygomatic arch using monocortical screws with lengths prescribed in accordance with the case plan.

The prosthetic component of the mandibular ramus is then placed (the previously planned size), and the submandibular incision area is irrigated profusely with saline and topical antibiotic solution. After confirming the adaptation of the prosthetic part of the mandibular ramus and the coincidence of the holes made with the surgical guide, one of the more cranial screws is placed without tightening it completely, and then one of the more caudal screws is placed. Next, the prosthetic head of the mandibular ramus is checked to confirm that it is located in the most posterosuperior part of the prosthetic fossa, and the two screws placed are tightened. Then, the rest of the screws are placed with the planned lengths so as not to damage the inferior alveolar nerve.

Once the fixation of the prosthesis has been completed, we can close the incisions before releasing the intermaxillary block only if we are certain that the 3D plan coincided with the surgical procedure. This will avoid cross-contamination if we return from the oral cavity to the articular surgical field.

The endaural and submandibular incisions are meticulously layered closed using 3/0 Vicryl and 5/0 monofilament sutures. The inferior border of the masseter muscle is sutured to the periosteum of the mandibular angle. Finally the incisions are covered with Steri-Strips™.

In the postoperative period, oral antibiotic therapy (amoxicillin/clavulanic acid 1 g/8 h) was prescribed for the first 7 days, along with analgesics, anti inflammatory drugs and elastic intermaxillary block.

### 2.4. Follow-up Visits

During the first two weeks the patient is checked every 4–5 days. The sutures are removed after about 10 days, and the dental occlusion is checked. Intermaxillary blocking is maintained with elastics to guide dental occlusion during the first weeks if necessary. During the first 3–4 weeks, only gentle opening and closing exercises are performed. Subsequently, the rehabilitator will prescribe exercises and complementary muscular treatments to improve the oral opening.

Aesthetic results: An aesthetic evaluation was performed by the patients to assess scores in facial symmetry, appearance of scars, and mandibular projection. The results were classified with scores 0 (“poor”), 1 (“fair”), and 2 (“good”). 

Functional results: Chewing and feeding were evaluated, and the results were classified with scores 0 (liquid diet), 1 (soft diet), and 2 (normal diet).

## 3. Results

Four patients followed for years for temporomandibular dysfunction and previously operated on by arthroscopy or open joint surgery that have been reconstructed with stock TMJ prostheses (STMJP) through virtual surgical planning (VSP). STL models with surgical and positioning guides were included. 

The follow-up period was from 11 months to 1 year 6 months (average 15 months); the median number of previous TMJ surgeries was two. The mean preoperative MIO was 24.6 mm and at longest follow-up was 36.4 mm. The median preoperative TMJ pain score was 8, and the median postoperative TMJ pain was 3. All patients have improved their mandibular function with a clear improvement of their initial situation.

All patients evolved uneventfully, and there were no infections signs. The immediate postoperative period was painless in all patients, with only slight discomfort. Partial reversible facial nerve palsy occurred in one of the four patients. No implant failure or screw loosening of the fossa or mandibular ramus components were observed.

All patients reported a good aesthetic result. In terms of functional results, speech articulation was evaluated as intelligible language in all patients. All patients reported a regular diet (Table 1).

### Case Presentation

A 36-year-old female patient came to our department with limited oral aperture, right open bite, and facial pain that was difficult to control.

**Figure 1:**
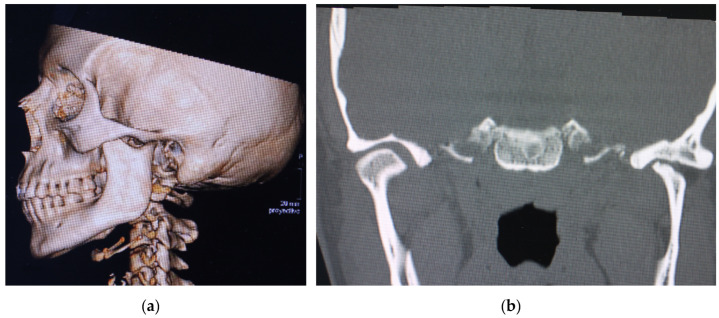
Preoperative computed tomography. (**a**) Lateral view; (**b**) flattening and degeneration of the left mandibular condyle.

She had been followed up in our practices since 2016 for left temporomandibular dysfunction, which had been operated on several times since then—left TMJ arthroscopy in May 2018 and left meniscectomy in October 2019. After 4 years of follow-up with good pain control and an oral opening of 37 mm at the last check-up, the patient returned to our clinic with left preauricular pain with a visual analogue scale (VAS) index of 8/10 and a decrease in oral opening to 25 mm. After orthopantomography and computed tomography, flattening of the left mandibular condyle, anterior osteophyte, and collapse of the joint space were observed (Figure 1a,b).

After confirming the clinical worsening of the left TMJ, it was decided to place a Walter–Lorenz stock prosthesis (Zimmer Biomet Inc., Biomet Microfixation, Jacksonville, FL, USA) with virtual surgical planning and surgical guides. A sagittal osteotomy of the right mandibular ramus was necessary to avoid excessive rotation of the right condylar while maintaining the planned occlusion.

**Figure 2:**
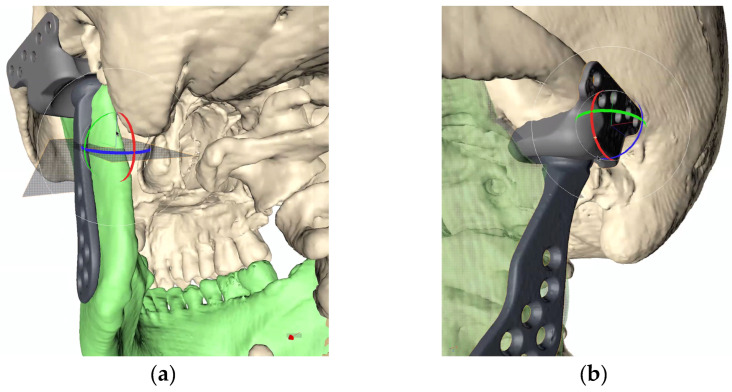
Virtual surgical planning. (**a**) Adaptation of the mandibular ramus component and planning of bone resection; (**b**) fitting of the glenoid component.

Virtual planning (Timeus^®^, CD Ortosan, Madrid, Spain) was performed for placement of a stock prosthesis. The 3D reconstruction was aligned with the STL files obtained from the patient’s intraoral scan and the diagnostic wax-up. After defining the mandibular position with the best possible dental occlusion, the osteotomies were planned according to the final position of the stock prosthesis both at the level of the fossa and at the level of the mandibular ramus (Figure 2a,b).

**Figure 3:**
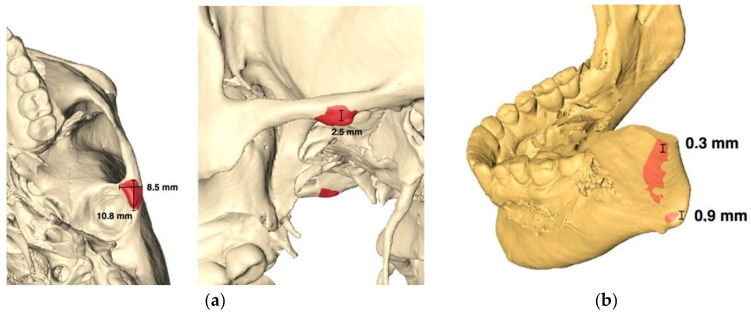
Adaptation of the prosthesis to the surface of the bone; (**a**) remodeling of the eminence. (**b**) Remodeling of the mandibular ramus. The remodeling area is shown in red.

This manufacturing company provides three distinct sizes for each of the parts, allowing surgeons to choose the one that aligns best with patient requirements. Following the selection of the appropriate size, a minor bone remodeling is also planned in the eminence and mandibular ramus areas to ensure optimal adaptation of the prosthesis to the bone surface. (Figure 3a,b).

**Figure 4:**
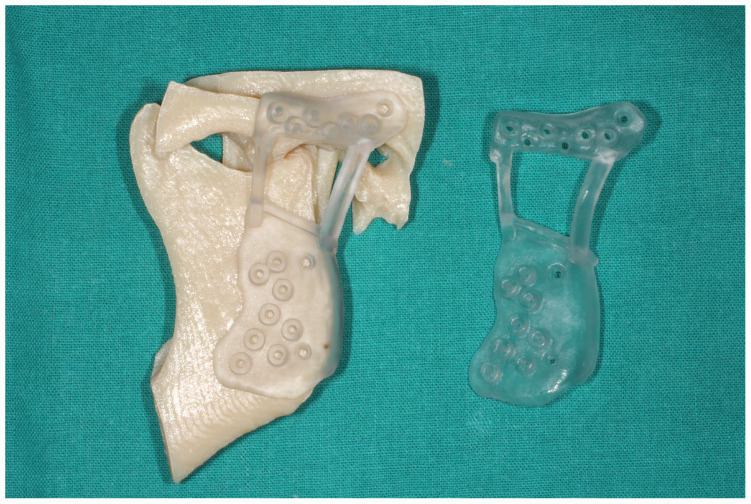
Stereolithographic model of the patient and cutting and positioning guides.

After checking the virtual planning, the cutting guides are designed, which will also be used to position the fixing screws for the prostheses. We will have a stereolithographic printed model before the surgical intervention where we will check the occlusal splint and the cutting guides and to serve as a reference for the surgery (Figure 4).

**Figure 5:**
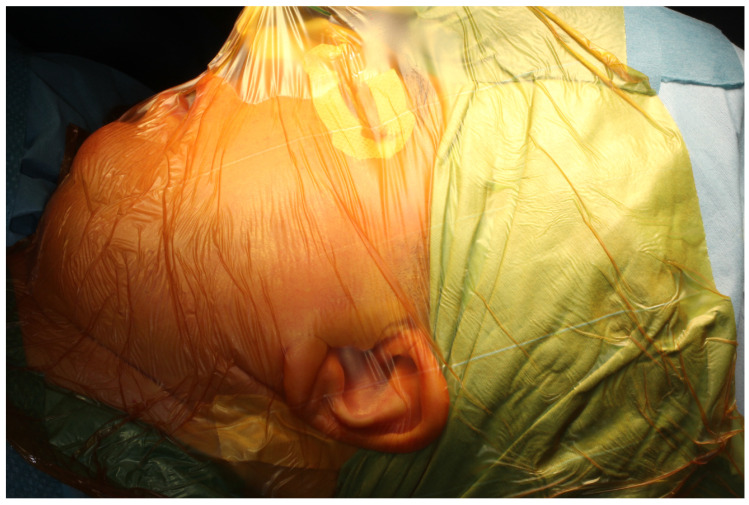
TMJ surgical field is isolated using Ioban^®^ adhesive drape.

The surgery was performed under general anesthesia and nasotracheal intubation. After infiltration with saline and vasoconstrictor (1:200.000 epinephrine) at the approach sites, an adhesive drape is applied. A 3M™ Ioban^®^ 2 antimicrobial adhesive drape is used to provide a sterile surface at the wound edge at the start of surgery and continuous antimicrobial activity throughout the procedure (Figure 5). After local infiltration with vasoconstrictor a preauricular and submandibular incisions are made for the approach to the joint and the mandibular ramus.

**Figure 6:**
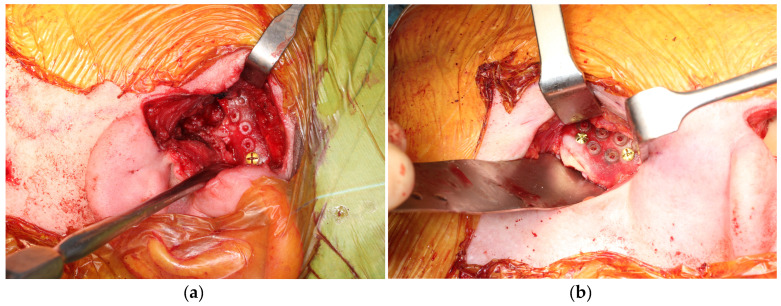
Intraoperative view. Cutting and positioning guides in fossa (**a**) and mandibular ramus (**b**).

Once the approach to the mandibular condylar and zygomatic arch has been made, the cutting and positioning guides for the prostheses are introduced (Figure 6a,b). At this point an intermaxillary block is applied in the dental occlusion that was performed.

**Figure 7:**
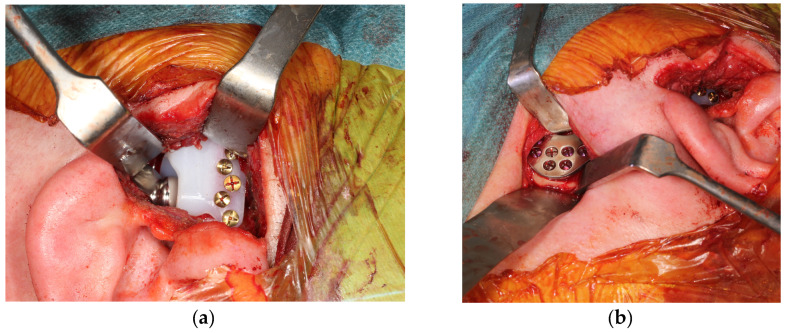
Intraoperative photographs. (**a**) Adaptation of fossa component. (**b**) Adaptation of condyle component.

After performing the osteotomies and marking the holes for the positioning of the prosthesis, the fossa part is inserted first, followed by the prosthetic part of the mandibular ramus, confirming the correct positioning of the prosthesis as described in the following section (Figure 7a,b). 

**Figure 8:**
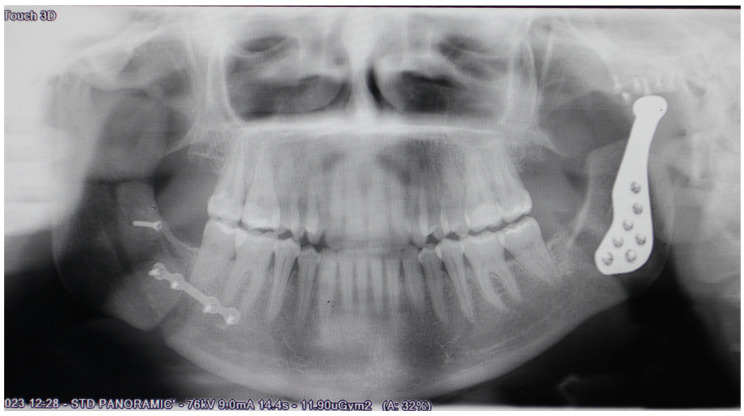
Follow-up orthopantomography (6 months) showing left total joint prostheses in place.

The length of each screw is established in the planning according to the osseous thickness of each area. The incisions of the approaches are closed and finally the dental occlusion is restored in the initial surgical splint. Finally, the sagittal osteotomy of the right mandibular ramus is performed and fixed with the occlusion that marks the final splint, verifying that it is similar to what was planned (Figure 8).

Antibiotic treatment is maintained during the first week and the stitches are removed after about 10 days. In the check-ups during the first month, the patient presented an oral opening similar to the previous one (30 mm) without pain and with a stable dental occlusion. Rehabilitation treatment was started around 4 weeks after the operation. At the 3-month check-up the patient had an oral opening of 34 mm and a VAS of 4/10, and a stable occlusion was maintained. At 6 months, the patient had an oral opening of 40.3 mm and a VAS of 3/10 with good mandibular function and stable occlusion.

## 4. Discussion

Temporomandibular joint (TMJ) replacement stands as a monumental advancement in the landscape of TMJ surgery, marking a pivotal moment in recent years [12,13]. Extensive literature corroborates the efficacy of both custom-made and stock devices, heralding a new era in the field. Presently, autologous reconstruction is predominantly relegated to pediatric patients or individuals grappling with the aftermath of failed joint prostheses.

The commendable attributes of custom-made prostheses extend beyond their superior adaptation to the anatomical region. Their efficacy lies in the realm of biomechanics, tailored to the nuances of each specific case, resulting in a diminished rate of micromovements. This not only fosters superior bone adaptation but also translates into heightened osseointegration, thereby bolstering the overall durability of these prostheses [1].

In the intricate domain of orthognathic surgery involving TMJ prostheses, precision is paramount. The zenith of bone fit and theoretical durability is epitomized by the deployment of custom-made prostheses [14]. Nevertheless, pragmatic considerations often necessitate a recourse to stock prostheses in certain hospital environments. The appeal of these lies in their cost-effectiveness and near-immediate availability, albeit at the expense of the tailored precision offered by their custom-made counterparts [15,16].

In the contemporary landscape, the integration of 3D virtual planning and the utilization of cutting and positioning guides for stock prostheses has revolutionized their adaptability. This not only mitigates economic costs but also facilitates immediate surgical interventions for patients experiencing acute pain and restricted jaw mobility, with the prostheses becoming available within days [10,12].

While stock prostheses may not be bespoke for individual patients, the advent of 3D virtual planning and the strategic use of cutting and positioning guides have augmented precision in the recipient anatomical area. This encompasses both the glenoid fossa and mandibular ramus, offering the flexibility to choose different component sizes for an optimal fit [17].

The meticulous planning of cutting guides is aimed at achieving the utmost adaptation of stock prostheses in the fossa and mandibular ramus components. Additionally, they incorporate strategically positioned holes for the final placement of prosthesis components, minimizing the risk of injury to the inferior alveolar nerve during drilling and screw placement.

Recent findings by Westermark based on an extensive 8-year follow-up underscore the reliability of stock prostheses, dispelling concerns regarding micromovements or incomplete adaptation to the bone surface [17]. Noteworthy challenges emerge from scarring resulting from prior surgical interventions, complicating dissection and elevating the risk of facial nerve interference. Some studies indicate an inverse correlation between treatment success after TMJ reconstruction and the number of prior surgical procedures [18,19].

The integration of intraoperative navigation, virtual planning, and surgical guides has emerged as a transformative force, enhancing the precision of surgery. This is particularly relevant in cases involving temporomandibular joint prostheses, especially when navigating anatomical variances [20].

In our comprehensive case series, a judicious blend of Biomet stock devices, requiring surgeon fitting at implantation, and custom-made devices, designed and manufactured with CAD/CAM techniques to match individual patient anatomy, was employed. This approach ensures a stock prosthesis adapted to each unique case, with the size chosen for an optimal fit and the ideal position in both the fossa and mandibular ramus [17].

Salter’s emphasis on proper post-surgical rehabilitation resonates profoundly in the recovery trajectory of patients undergoing TMJ prostheses [21]. Physiotherapy post-surgery proves indispensable in preventing adhesion formation, mitigating soft tissue scarring, and stimulating normal muscle function [22]. However, a critical juncture arises when patients, having achieved an acceptable oral opening, may inadvertently slacken their commitment to prescribed exercises. Therefore, it becomes imperative for the physiotherapist to vigilantly follow the patient for at least the initial 6 months post-surgery.

Regrettably, a substantial portion of patients undergoing TMJ prostheses in our cases present a challenge. Many are chronic users of analgesics pre-surgery, often with a history of previous joint surgeries. As highlighted by Aagard and Thygesen, these individuals necessitate specialized care from multidisciplinary pain units after surgery [23]. The complexity of managing chronic pain in such cases requires a nuanced approach, underscoring the importance of an integrated and comprehensive care continuum.

## 5. Conclusions

The landscape of TMJ surgery has undergone a paradigm shift with the advent of TMJ replacement, offering a spectrum of options ranging from custom-made to stock devices. The nuanced choice between these options hinges on a delicate balance between precision, cost considerations, and logistical constraints. The integration of cutting-edge technologies like 3D virtual planning and surgical guides has not only expanded the horizons of stock prostheses but has also paved the way for immediate interventions in cases of acute pain and limited jaw mobility.

While the pursuit of precision through custom-made prostheses remains ideal, pragmatic considerations often lead to the adoption of stock devices. The key lies in striking a balance between tailored precision and practical feasibility. The challenges posed by scarring and prior surgical interventions underscore the importance of meticulous planning and intraoperative technologies to navigate these complexities successfully.

As the field continues to evolve, the multidisciplinary approach, encompassing surgical expertise, rehabilitation protocols, and pain management strategies, will play a pivotal role in optimizing patient outcomes. The delicate interplay between surgical innovation and postoperative care will define the trajectory of TMJ surgery, offering hope and improved quality of life for individuals grappling with temporomandibular joint disorders.

In conclusion, we believe that stock TMJ prostheses with virtual surgical planning and surgical guides are a good alternative for TMJ reconstruction at the present time. Recent reports and our experience show good results in cases where the mandibular anatomy is not altered and in cases with little mandibular asymmetry. Nonetheless, prospective and randomized trials are required with long-term follow up to assess their performance and safety.

## Figures and Tables

**Table 1:** Descriptive variables in all patients. MIO (maximal interincisal openig). VAS (visual analogic scale).

Gender/Age (Years)	Image	MIO Preop (mm)	MIO at 6 Months (mm)	VAS Preop	VAS at 6 Months	Functional Result	Aesthetic Result	Complications
**F/36**	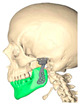	25.9	36.4	8	3	2	2	None
**F/49**	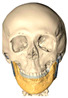	15.6	35.4	9	4	2	2	Partial left facial palsy
**F/68**	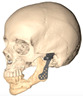	30.5	40.3	8	3	2	2	None
**F/42**	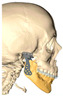	26.4	33.5	7	2	2	2	None
**Average**		24.6	36.4	8	3	2	2	

## Data Availability

The data presented in this study are available on request from the corresponding author. The data are not publicly available due to data protection regulations.
